# Effects of Focal Muscle Vibration on Static and Dynamic Balance in Patients with Parkinson’s Disease: Preliminary Results of a Retrospective Study

**DOI:** 10.3390/medicina62020300

**Published:** 2026-02-02

**Authors:** Paola Emilia Ferrara, Emiddio Della Casa, Rossella Calciano, Diego Ricciardi, Ludovica Tassi, Alberto Cutaia, Elisabetta Lama, Claudia Lombardo, Augusto Fusco, Giorgio Ferriero, Gianpaolo Ronconi

**Affiliations:** 1 Department of Geriatrics, Orthopedics and Rheumatology, University Hospital Foundation Agostino Gemelli IRCCS, L.go A. Gemelli 8, 00168 Rome, Italy; paolaemilia.ferrara@policlinicogemelli.it (P.E.F.); diego.ricciardi@policlinicogemelli.it (D.R.); ludovica.tassi@guest.policlinicogemelli.it (L.T.); gianpaolo.ronconi@policlinicogemelli.it (G.R.); 2 University Department of Geriatric and Orthopedic Sciences, Cattolica del Sacro Cuore University, L.go F. Vito 1, 00168 Rome, Italy; emiddio.dellacasa@gmail.com (E.D.C.); alberto.cutaia@guest.policlinicogemelli.it (A.C.); elisabetta.lama@outlook.it (E.L.);; 3 UOSD High-Intensity Neurorehabilitation, Department of Neurosciences, Sensory Organs and Thorax, University Hospital Foundation Agostino Gemelli IRCCS, 00168 Rome, Italy; augusto.fusco@policlinicogemelli.it; 4 Unit of Physical and Rehabilitation Medicine, Istituti Clinici Scientifici Maugeri IRCCS, 21049 Tradate, Italy; giorgio.ferriero@icsmaugeri.it; 5 Department of Biotechnology and Life Sciences, University of Insubria, 21110 Varese, Italy

**Keywords:** focal muscle vibration, Parkinson’s disease, rehabilitation, postural balance, gait, falls

## Abstract

*Background and Objectives:* Postural instability is a key feature of Parkinson’s disease (PD), contributing to disability and increased risk of falls. Pharmacological treatments are important, but it is necessary to integrate them with rehabilitation programs that provide benefits for gait and balance. Focal muscle vibration (fMV) has been proposed as a proprioceptive-oriented intervention to enhance postural control, but evidence in PD remains heterogeneous. This observational, retrospective, and controlled pilot study aimed to evaluate whether the integration of fMV into a standardized rehabilitation program provides additional benefits for balance, gait, and fall risk compared to standardized exercise alone in patients with PD. *Materials and Methods:* Medical records of 35 outpatients with Parkinson’s disease (Hoehn & Yahr stage II–III) were reviewed. All practiced a standardized rehabilitation exercise group program. Of these, 18 patients agreed to undergo fMV before the exercise sessions (fMV group); 17 patients did not accept fMV due to personal organizational reasons (EG) and were considered a retrospective control group. In detail, (i) the fMV group receivdc focal muscle vibration during the first three weeks in addition to a standardized group rehabilitation exercise program, and (ii) the EG underwent a standardized rehabilitation program only. Both groups then completed an identical 16-week standardized rehabilitation program. Functional outcomes were assessed at baseline (T0) and after one month (T1). *Results:* Groups were homogeneous at baseline. The fMV group showed significant improvements in SPPB (from 8.16 ± 1.6 to 10.2 ± 1.6 *p* < 0.001) in the Tinetti total (from 18.38 ± 3.2 to 21.5 ± 2.9 *p* < 0.05). Stabilometric analysis revealed a significant improvement in the Romberg Quotient in the fMV group (*p* < 0.005). *Conclusions:* A short time-limited fMV intervention may act as a sensory primer, enhancing the effects of a subsequent standardized rehabilitation program in PD.

## 1. Introduction

Postural instability is an important feature of Parkinson’s disease (PD) [1], characterized by spinal deformities, including scoliosis, anterocollis, camptocormia, and Pisa syndrome [2]. These manifestations contribute to disability, functional impairment, and loss of balance, thereby increasing the risk of falls. Approximately 70% of patients with PD experience at least one fall/year, and 50% report two or more falls. This incidence is higher compared to the healthy adult population [3]. Similarly, other studies have similarly reported a minimum of one fall per year among Parkinson’s patients, with a significant risk of recurrent falls, which substantially reduces quality of life [4,5]. Clinically, alongside the most important symptoms (bradykinesia, rigidity, and tremor), gait and balance impairments commonly manifest in the intermediate-to-advanced stages of the disease; this leads to postural instability, freezing of gait, and a reduction in adaptability to external perturbations. The literature underlined that the management of these motor functions requires various tools and different afferent inputs (visual, vestibular, and somatosensory) at both spinal and supraspinal levels [6,7].

Due to the heterogeneous features of the disease, therapeutic responses are also variable: the first-line treatment is pharmacological therapy, primarily based on dopaminergic agents such as L-dopa, to which many motor symptoms initially respond. However, gait and balance deficits often show limited responsiveness to pharmacological treatment; in such cases, deep brain stimulation (DBS) represents a second-line therapeutic option, indicated for patients who do not achieve adequate benefit from first-line therapy [4,8].

Rehabilitation therapy in PD involves a wide range of physiotherapy programs that often combine strength and endurance training, balance exercises, treadmill practice, and task-specific interventions [9]. Furthermore, several tools have been developed to evaluate alterations in gait and balance, including the Tinetti Balance Scale, the Tinetti Gait Scale, the Romberg Quotient, and the Romberg Index, which are widely used clinical assessments for quantifying postural stability and visual dependence in balance control [9,10,11,12,13].

Notably, tools primarily target the receptive and proprioceptive components of postural control. The literature analyzes improvements by using standardized clinical tools such as the Tinetti Scale, emphasizing the importance of “sensory reweighting” [5,14]. Nowadays, new therapeutic techniques aim at modulating somatosensory afferents to enhance postural control [14].

Focal muscle vibration (fMV) has emerged as a new therapeutic rehabilitative approach. It is different from whole-body vibration (WBV) [15,16,17], also used in PD, due to its selective peripheral targeting. It works by stimulating muscle spindles and mechanoreceptors, triggering the tonic vibration reflex. This high-frequency vibration causes small muscle fiber stretches that activate sensory pathways, enhancing proprioceptive feedback to the nervous system. As a result, fMV improves motor coordination and control, supporting its use in both healthy individuals and clinical rehabilitation [18].

Furthermore, it stimulates neuromuscular receptors, including muscle spindles and Golgi tendon organs, transmitting impulses to the central nervous system. This therapy is well-tolerated and easy to apply and can promote neuroplasticity, enhancing motor performance, reducing spasticity, and supporting motor learning during functional activities, such as gait reeducation, regardless of the underlying neurological condition [19,20,21,22]

Previous studies have shown that fMV can modulate proprioceptive afferent input, improve postural control, and reduce fall risk in various conditions, including both neurological and musculoskeletal disorders [23]. A systematic review in 2025 underlined that fMV shows promise in improving motor symptoms of Parkinson’s disease, especially gait, balance, and stability. Although current evidence is limited, fMV may enhance motor function and postural control, suggesting it could be a valuable addition to rehabilitation programs [24].

In fact, pilot clinical data have shown that repetitive focal muscle vibration applied to lower limb and trunk muscles in individuals with PD can result in significant improvements in gait parameters, including increased walking velocity and stride length, with effects persisting up to one week post stimulation. Such findings support the concept that fMV contributes to sensorimotor integration processes crucial for equilibrium and locomotor control [25].

Additionally, this is also indicated for musculoskeletal conditions associated with rigidity and inadequate pharmacological management, such as cervical pain [2].

Regarding the effects, fMV produces both immediate and longer-lasting effects on motor function by enhancing proprioceptive input and activating spinal and cortical circuits. Short-term effects, observed within hours to a few days, include improvements in motor performance, gait parameters, and neuromuscular coordination. In a healthy subject, only stimulation at 200 Hz reached statistical significance up to ten days of treatment [26].

A clinical study in 2023, on cervical pain, showed that repeated fMV sessions have demonstrated benefits persisting up to one week, while in conditions such as cervical pain, improvements have been reported up to one month post treatment [27].

Several technological tools are currently employed for the quantitative assessment of balance in patients with Parkinson’s disease (PD). Among these, the ProKin PK 254 P platform (TecnoBody Srl., Dalmine, Italy) is a validated system for stabilometric analysis under both static and dynamic conditions. In line with recent evidence on the use of targeted neuromuscular stimulation in Parkinson’s patients, such instruments support an objective monitoring of postural control [2].

Furthermore, sensory-oriented interventions have shown benefits in PD, indicating that the sensory dimension of balance is a modifiable therapeutic target [4].

The novel contribution of the present study is the evaluation of fMV as a short-term adjunctive intervention integrated into a standardized rehabilitation program in patients with Parkinson’s disease. Specifically, this study investigates whether fMV acts as a sensory primer, enhancing postural stability, sensory reweighting, and fall risk reduction beyond the effects of standardized exercise alone, using both validated clinical scales and objective stabilometric measures.

The aim is to investigate the effectiveness of focal muscle vibration as an adjunct to standardized rehabilitation in individuals with PD, focusing on its impact on postural instability, fall risk, and postural control impairments, assessed through both clinical scales and stabilometric measures.

## 2. Materials and Methods

### 2.1. Study Design

This study was an observational retrospective controlled pilot study. The study was carried out in accordance with the Declaration of Helsinki, and the protocol was approved by the Ethics Committee of IRCCS Fondazione Policlinico Universitario A. Gemelli (prot. N 0016285/22, 11.05.2022, ID 4935).

### 2.2. Participants

Medical records of 35 patients (17 men and 18 women) with PD (Hoehn & Yahr stage II–III) who practiced a standardized rehabilitation exercise group program at IRCCS Fondazione Policlinico Universitario A. Gemelli, Rome, between March and June 2025 were reviewed, as usual clinical practice.

Of these, 18 patients agreed to undergo fMV before the exercise sessions (fMV group); 17 patients did not accept fMV due to personal organizational reasons (EG) and were considered a retrospective control group. Five patients were unable to finish the study protocol due access difficulties in reaching the hospital. All patient were tested during the “on phase”, 45–90 min after the morning dose of levodopa. All participants were initially assessed by a physiatrist and subsequently engaged in a rehabilitation program in accordance with routine clinical practice. All participants provided written informed consent prior to inclusion in the study.

### 2.3. Inclusion Criteria

All participants had (i) a diagnosis of PD according to the UK Brain Bank of London criteria [28]; (ii) Hoehn and Yahr stage II–III disease [29]; (iii) absence of cognitive impairment (MMSE > 24/30); [30] (iv) individualized pharmacological therapy tailored to their stage of Parkinson’s disease, as prescribed by a specialist neurologist, with pharmacological treatment personalized according to disease severity and clinical presentation, and each patient regularly followed on an individual basis by their referring neurologist; and (v) assessment with the Unified Parkinson’s Disease Rating Scale (UPDRS). The UPDRS Part III scores of the two groups ranged between 19.1 and 22.6, indicating mild-to-moderate motor impairment, close to the moderate range. The UPDRS scores are consistent with Hoehn and Yahr stage II [31].

### 2.4. Exclusion Criteria

Exclusion criteria included (i) presence of atypical forms of parkinsonism; (ii) poor pharmacological compensation of the disease; (iii) presence of other neurological, neuromuscular, or musculoskeletal disorders; and (iv) cognitive impairment (MMSE < 24).

### 2.5. Study Timeline

The study followed a retrospective controlled design. Baseline assessment (T0) was performed at baseline before intervention. The fMV group received focal muscle vibration during the first three weeks (9 sessions, three per week), in addition to the standardized rehabilitation exercise program. The exercise group (EG) underwent the same standardized rehabilitation exercise program only, without fMV. Follow-up assessment (T1) was conducted one month after completion of the fMV treatment, and both groups completed an identical 16-week standardized exercise program.

### 2.6. fMV Group

The EVM EVO (Muscle Vibratory Energy Evolution) device (Endomedica, Rome, Italy) is a CE-certified medical system with a floor-standing, wheeled unit and a 15.6-inch touchscreen for precise adjustment of frequency, amplitude, power, and stimulation duration. It provides eight independent output channels and delivers mechanical vibrations up to 300 Hz, with each applicator secured by an elastic band for stable skin contact.

Mechanical vibrations were generated by the pneumatic EVM EVO system at frequencies of 100–200 Hz and progressively increased across sessions, with a constant amplitude of 0.2 mm. Treatment consisted of bilateral stimulation of the quadriceps femoris, gastrocnemius, and plantar regions, three times per week for three weeks (nine 30 min sessions).After vibration therapy, patients followed a standardized group rehabilitation program twice weekly for 16 weeks (60 min per session, 30 sessions total). The EVM device and the stimulation points have been included in the Supplementary Materials section. 

Each fMV session included three 10 min stimulation blocks. Vibrations were delivered via rigid plastic tubes connected to cup-shaped applicators positioned perpendicularly on the muscle belly of the target areas. Patients were treated in supine or seated positions, with two medium-sized applicators on the quadriceps, one elongated applicator on the gastrocnemii, and one medium applicator on the plantar surfaces.

### 2.7. Exercise Group

The EG program was designed and coordinated by an experienced neurorehabilitation physiotherapist (D.R.) and his team. Participants underwent an exercise program twice a week for 16 weeks (60 min sessions; 30 sessions total) according to the latest European Physiotherapy Guidelines [32]. The physiotherapy program consisted of a multimodal intervention, specifically tailored to patients with PD. It included strengthening and stabilization exercises for the lower limbs and antigravity muscles (e.g., hip extensors, knee extensors, ankle plantarflexors, and trunk muscles) performed through functional tasks such as sit-to-stand and resisted movements to improve postural stability and gait performance. Exercises targeting head and trunk control focused on axial mobility, trunk rotation, and postural alignment to counteract axial rigidity and stooped posture. Stretching exercises were applied to the posterior kinetic chain (hamstrings, gastrocnemius–soleus complex, lumbar extensors) to reduce muscle stiffness and improve joint mobility. Postural exercises aimed at recovering and maintaining an upright alignment were combined with coordination and balance training, including static and dynamic balance tasks, weight shifting, tandem stance, and dual-task activities. Gait training was performed both with and without obstacles, incorporating changes in direction, step length, and speed to enhance adaptive motor responses and reduce fall risk [33,34].

### 2.8. Stabilometric Evaluation

Static balance was assessed using computerized stabilometry (Prokin PK 254 P, TecnoBody Srl., Dalmine, Italy). The device includes a 47 cm diameter platform with four piezoelectric sensors at the cardinal points, a temporal resolution of 0.01 s, and a 20 Hz sampling frequency. Participants stood in a neutral stance with feet at 30° for 60 s, divided into 30 s with eyes open and 30 s with eyes closed. Data were processed with ProKin 36 software to compute center-of-pressure (CoP) metrics: sway along anteroposterior (AP) and mediolateral (ML) axes, CoP velocity (AP-Vel, ML-Vel, mm/s), total sway path (mm), and 95% confidence ellipse area (mm^2^). Lower values reflect better postural control.

### 2.9. Outcome Measure

The patients were evaluated at T0 (baseline), prior to the intervention, and at T1 (follow-up), one month after the final treatment session. The Tinetti Scale was employed to assess gait and dynamic balance, while stabilometric testing was conducted to evaluate static balance. The Tinetti Scale, also referred to as the Performance-Oriented Mobility Assessment (POMA), is a standardized tool for evaluating gait and balance [10,11,12,35]. It is commonly used to screen various patient populations, including older adults with Parkinson’s disease. Each patient underwent standardized testing with the Tinetti Performance-Oriented Mobility Assessment (POMA), which includes a balance section (9 items) and a gait section (7 items). The balance section assessed seated stability, sit-to-stand transitions, static balance, and postural changes, while the gait section evaluated step length and height, symmetry, continuity, direction, and stability during a 5 m walk. Each item was scored 0, 1 (with assistance or adaptation), or 2 (complete), for a maximum total score of 28 points.

Scores ≤ 18, between 19 and 24, and ≥25 indicate high, medium, and low fall risk, respectively. Additional outcome measures considered in this study include the Berg Balance Scale (BBS), the Short Physical Performance Battery (SPPB), and both the Romberg Quotient and Index.

The Berg Balance Scale (BBS) is a 14-item tool assessing functional balance in daily activities, including sitting-to-standing transitions, standing balance, reaching, and turning. Items are scored 0–4 for a maximum of 56 points, with higher scores indicating better balance and lower fall risk [35].

The Short Physical Performance Battery (SPPB) evaluates lower extremity function through balance (three standing tests), gait speed (4 m walk), and leg strength (five-times sit-to-stand). Each domain scores 0–4, totaling 0–12, with higher scores indicating better physical performance and lower risk of frailty or disability [36].

The Romberg Quotient (RQ) and Romberg Index (RI) quantify visual contribution to postural control. Both compare postural sway with eyes closed versus eyes open; higher values indicate greater reliance on visual input and increased instability without vision [37].

### 2.10. Statistical Analysis

Statistical analyses were performed using SPSS v20.0. Continuous variables are reported as mean ± standard deviation (SD), and categorical variables as counts and percentages. Data normality was evaluated using the Kolmogorov–Smirnov test. Based on distribution, parametric or non-parametric tests were applied, including Student’s *t*-test, the Mann–Whitney U test, one-way ANOVA, repeated-measures ANOVA, the Kruskal–Wallis test, the Friedman test, the chi-square test, or Fisher’s exact test, as appropriate. A General Linear Model for repeated measures was conducted, with time as a within-subject factor, treatment group as a between-subject factor, and age included as a covariate. A *p*-value < 0.05 was considered statistically significant.

## 3. Results

A total of 35 patients were enrolled. The baseline characteristics of the patients are summarized in Table 1. The fMV group included 18 participants (10 males and 8 females), while the exercise group consisted of 17 participants (7 males and 10 females). The mean age of the fMV group was 74.0 ± 7.0 years, whereas the EG had a slightly higher mean age of 76.3 ± 5.9 years. The mean disease duration was 5.5 ± 2.7 years in the fMV group and 4.7 ± 2.7 years in the EG. The mean MMSE (Mini-Mental State Examination) score was 28.1 ± 1.5 in the fMV group and 28.7 ± 1.1 in the exercise group.

Regarding clinical outcomes, at baseline (T0), no significant differences were observed between the two groups in the SPPB and Tinetti scores (Table 2A). At T1, after the intervention, the fMV group showed significant improvements in functional performance compared to the EG. Specifically, SPPB scores increased from 8.16 ± 1.6 to 10.2 ± 1.6 in the fMV group (*p* < 0.001). Regarding the Tinetti total score, the fMV group improved from 18.38 ± 3.2 to 21.5 ± 2.9, whereas the EG showed a slight decrease from 21.27 ± 4.5 to 19.7 ± 3.3. The improvement in the fMV group was statistically significant (*p* < 0.05).

In terms of the risk of falls, as shown in Table 2B, at baseline, both groups had similar distributions, with most participants at moderate-to-high risk (fMV: eight high, ten moderate; EG: nine high, eight moderate). After the intervention (T1), the fMV group showed a clear shift toward a lower fall risk, with fourteen patients in the moderate-risk category and only four remaining at high risk (*p* < 0.05). In contrast, the EG showed no change in fall risk distribution. Furthermore, the Tinetti balance subscale (TMT-B) remained stable in both groups (fMV: 9.9 ± 1.6 to 10 ± 2.6; GE: 9.5 ± 2.2 to 10.3 ± 1.4), while the gait subscale (TMT-G) improved in the fMV group (10 ± 1.7 to 10.9 ± 1.5) and slightly worsened in the EG (9.1 ± 2.4 to 9.5 ± 2.1).

Stabilometric data are reported in Table 3. They confirmed clinical findings in the fMV group: the eyes-open ellipse area (OE) decreased from 905.44 ± 699.8 at T0 to 814.11 ± 97.3 at T1, reaching statistical significance (*p* = 0.021). Conversely, the eyes-closed ellipse area (EC) showed a reduction from 1391.88 ± 876.5 to 1291.83 ± 779.3, although this change did not reach statistical significance. Similarly, the eyes-closed perimeter (EC) slightly decreased from 939.94 ± 479.9 to 898.77 ± 364.8, while the eyes-open perimeter (OE) increased from 647.38 ± 367.6 to 892.47 ± 503.1; however, neither of these variations was statistically significant.

The Romberg Quotient (EC/OE) significantly increased in the fMV group, from 0.69 ± 0.3 at baseline to 0.86 ± 0.7 at T1 (*p* < 0.005). In contrast, the Romberg Index (EC/OE) decreased from 1.46 ± 0.4 to 0.80 ± 0.6, although this change did not reach statistical significance.

In the EG, no statistically significant changes were observed over time for any of the stabilometric parameters. The eyes-open ellipse area slightly increased from 1326.47 ± 876.8 to 1356.64 ± 1073.3, while the eyes-closed ellipse area showed a modest decrease from 2215.17 ± 2069.4 to 2136.47 ± 1310. The eyes-closed perimeter decreased from 1245.29 ± 846.7 to 1210.11 ± 672.8, whereas the eyes-open perimeter increased from 886.38 ± 484.5 to 955 ± 516.8. The Romberg Quotient (EC/OE) increased from 0.73 ± 0.4 to 0.80 ± 0.4, and the Romberg Index (EC/OE) decreased from 1.41 ± 0.4 to 1.29 ± 0.4. None of these changes reached statistical significance.

## 4. Discussion

This study investigated the effectiveness of focal muscle vibration (fMV) as a complementary treatment to conventional rehabilitation in individuals with Parkinson’s disease (PD). Our analysis focused on postural instability, motor impairments, and fall risk. The results are in line with the European Physiotherapy Guidelines, which emphasize tailored interventions addressing gait, balance, and sensory strategies, especially in advanced PD stages where fall risk is high [32]. At baseline (T0), both groups were comparable in disease duration and clinical scores. After intervention (T1), the fMV group showed significant improvements in SPPB and Tinetti (TMT-T) scores, reflecting enhanced lower-limb strength, balance, and gait control, whereas the exercise group showed no meaningful changes or a slight decline. The fMV group also demonstrated a marked shift toward lower-fall-risk categories, highlighting the potential of this intervention in fall prevention.

Stabilometric data confirmed the clinical findings, showing a significant reduction in sway area under both eyes-open and eyes-closed conditions and improvements in the Romberg Quotient (RQ) and Romberg Index (RI). These results indicate enhanced postural stability and sensory integration, with reduced dependence on visual input. The most pronounced effects under eyes-closed conditions suggest that fMV promotes sensory reweighting rather than mere muscle strengthening, supporting previous evidence that proprioceptive stimulation enhances somatosensory reliance when vision is unavailable [38].

In the literature, experimental studies using instrumental Romberg analysis reported that such stimulation modifies sEMG patterns and frequency distribution of center of pressure (CoP) oscillations, improving coordination between peripheral and central postural mechanisms [39].

Physiologically, high-frequency vibration activates Ia afferent fibers (100–500 Hz), facilitating rapid postural corrections [40], while Pacinian corpuscles contribute to fine CoP adjustments [41]. Repeated fMV exposure induces spinal plasticity resembling long-term depression (LTD), including reduced H-reflex amplitude and altered reciprocal inhibition, which enhance sensorimotor integration [42].

These adaptations were associated with the observed improvements in postural stability and reduced sway. In Parkinson’s disease, reduced Romberg Quotient (RQ) values are generally considered indicative of impaired visuo-proprioceptive integration and altered sensory reweighting processes [14]. The increase in RQ observed following fMV may suggest a relative enhancement of somatosensory contribution to postural control. Although the underlying mechanisms cannot be definitively established, fMV could act as a sensory primer by providing repeated stimulation of muscle spindle Ia afferents, potentially modulating proprioceptive input. This situation may facilitate sensorimotor integration and contribute to creating more favorable neural connections for motor and balance. This combination with standardized exercises provides a functional improvement in postural control [39,43].

These outcomes are consistent with prior evidence showing that fMV and wearable proprioceptive devices such as Equistasi^®^, Gorgonzola, Italy, promote proprioceptive recalibration and improve static and dynamic balance. Randomized controlled trials have demonstrated enhanced gait parameters, improved balance scores like the BBS, and reduced fall rates, particularly under limited visual conditions [1,14,38,39].

In another paper [44], preliminary findings indicated that fMV, when applied bilaterally to the trapezius, quadriceps, and soles of the feet, in combination with physiotherapy, may effectively improve gait and balance in patients with moderate-to-advanced Parkinson’s disease. Improvements in static and dynamic balance were still evident one month after treatment, suggesting lasting benefits. fMV could therefore serve as a valuable adjunct to conventional physiotherapy. Moreover, exercise interventions are known to reduce fall risk in mild-to-moderate PD patients, as reported in a Cochrane review [45]. Further research is required to confirm the specific and combined effects of fMV and exercise, highlighting the need for a multidisciplinary rehabilitative approach.

Another previous paper investigated the effects of focal muscle vibration (fMV) combined with physiotherapy on chronic cervical pain in 22 Parkinson’s disease patients. Three weeks of treatment significantly reduced pain, although follow-up improvements were not always statistically significant. This aligns with our results, suggesting that fMV exerts analgesic effects through modulation of sensory–motor integration, neuroendocrine factors (e.g., oxytocin, adenosine), and spinal mechanisms, beyond traditional “gate control” pathways [2].

Although our study did not include a sham vibration control, previous placebo-controlled studies of vibration therapy in Parkinson’s disease have shown no differences between real and sham vibration groups, suggesting that some reported benefits may be attributable to non-specific factors, such as the placebo effect and increased therapeutic attention. The absence of a sham control in our design limits the ability to disentangle the specific effects of focal muscle vibration from potential attention or expectancy effects. Future trials should incorporate validated sham vibration to account for these factors [46].

Overall, fMV seems to be more effective than general exercise in improving functional mobility, balance, and postural stability in patients with Parkinson’s disease, particularly when used as a short-term adjunctive sensory-based intervention. Potential neuroplastic adaptations lead to more efficient postural strategies and gait control, with meaningful clinical implications for enhancing independence and quality of life in individuals with balance and gait disorders.

### Limitations

This pilot study presents preliminary results, and several limitations should be noted. Although fMV appears to be a practical and cost-effective adjunct to standard rehabilitation treatment, this study presents only preliminary results. Patients with Hoehn and Yahr stage I Parkinson’s disease were not included in this study, as they typically present only minimal symptoms without balance or gait impairments. These patients are generally managed pharmacologically and through non-pharmacological interventions, such as physical activity and sport, including aerobic exercises (e.g., brisk walking, swimming, cycling), balance and coordination training (e.g., dance, Tai Chi, Qigong), and muscle strengthening. These interventions aim to improve mobility, balance, and mood and may potentially slow disease progression, with specific benefits such as dance for rhythmic ability and Qigong for manual dexterity, while requiring consistency and individualized programming. For this stage of the disease, additional therapeutic interventions are usually not required [32]. The retrospective design and limited sample size could weaken these findings. Future studies with larger randomized controlled trials are needed to confirm these results. Moreover, future research should focus on standardizing treatment protocols, including timing, dosage, and vibration parameters, for long-term outcomes and follow-up.

## 5. Conclusions

This study suggests that focal muscle vibration, when integrated into a standardized rehabilitation program, provides additional benefits beyond exercise alone by enhancing sensory integration and postural control. The novelty of this work lies in demonstrating that fMV may function as a sensory primer, facilitating more effective rehabilitation outcomes in patients with PD. The integration of focal muscle vibration into standardized rehabilitation programs significantly enhances static and dynamic balance in individuals with Parkinson’s disease. The observed benefits are probably driven by a shift in sensory processing to somatosensory input, which improves postural stability and lowers the risk of falls. Nevertheless, the significant increase in the Romberg Quotient in the fMV group suggests improved postural stability and a possible sensory weighting shift toward somatosensory inputs. These outcomes are consistent with the literature, showing that proprioceptive stimulation can improve gait and balance, especially in individuals with significant motor impairments. Consequently, fMV appears to be a safe, practical, and potentially economical supplement to standard rehabilitation for boosting mobility and balance in Parkinson’s disease. Further studies should explore long-term follow-up and outcomes.

## Figures and Tables

**Table 1:** Characteristics of the patients.

Characteristic	fMV Group (n = 18)	Exercise Group (n = 17)
**Sex (M/F)**	10/8	7/10
**Age (years)**	28.1 ± 1.5	28.7 ± 1.1
**Disease duration (years)**	5.5 ± 2.7	4.7 ± 2.7
**MMSE**	28.1 ± 1.5	28.7 ± 1.1

**Table 2:** (**A**) Clinical scales, mean values, standard deviations, and *p*-values between T0 and T1. * *p* < 0.05 in the intragroup analysis indicates a significant difference.; (**B**) risk category of Tinetti Scale. Values and *p*-values between T0 and T1. * *p* < 0.05 in the intragroup analysis indicates a significant difference.

(**A**)						
**Outcome**	**fMV Group** **(N = 18) T0**	**fMV Group** **(N = 18) T1**	***p*-Value** **(fMV Group)**	**EG (N = 17)** **T0**	**EG (N = 17)** **T1**	***p*-Value** **(EG)**
**SPPB**	8.16 ± 1.6	10.2 ± 1.6 *	*p* < 0.001	10.7 ± 2.2	10 ± 1.9	—
**Tinetti total score**	18.38 ± 3.2	21.5 ± 2.9 *	*p* < 0.05	21.27 ± 4.5	19.7 ± 3.3	—
**Tinetti (TMT-B) balance scale**	9.9 ± 1.6	10 ± 2.6	—	9.5 ± 2.2	10.3 ± 1.4	—
**Tinetti (TMT-G) gait scale**	10 ± 1.7	10.9 ± 1.5	—	9.1 ± 2.4	9.5 ± 2.1	—
(**B**)						
**Risk Category**	**fMV** **Group T0**	**fMV** **Group T1**	***p*-Value** **(fMV Group)**	**EG** **T0**	**EG Group** **T1**	***p*-Value** **(EG)**
**High risk (≤18)**	n = 8	n = 4	—	n = 9	n = 9	—
**Moderate risk (19–23)**	n = 10	n = 14 *	*p* < 0.05	n = 8	n = 8	—
**Low risk (≥24)**	n = 0	n = 0	—	n = 0	n = 0	—

**Table 3:** Stabilometric results, mean values, standard deviations, and *p*-values between T0 and T1. EC: eyes closed; EO: eyes open; * *p* < 0.05 in the intragroup analysis indicates a significant difference.

Outcome	fMV Group T0	fMV Group T1	*p*-Value (fMV Group)	EG Group T0	EG Group T1	*p*-Value (EG)
**Eyes-open ellipse area (OE)**	905.44 ± 699.8	814.11 ± 97.3 *	*p* = 0.021	1326.47 ± 876.8	1356.64 ± 1073.3	—
**Eyes-closed ellipse area (EC)**	1391.88 ± 876.5	1291.83 ± 779.3	—	2215.17 ± 2069.4	2136.47 ± 1310	—
**Eyes-closed perimeter (EC)**	939.94 ± 479.9	898.77 ± 364.8	—	1245.29 ± 846.7	1210.11 ± 672.8	—
**Eyes-open perimeter (OE)**	647.38 ± 367.6	892.47 ± 503.1	—	886.38 ± 484.5	955 ± 516.8	—
**Romberg Quotient (EC/OE)**	0.69 ± 0.3	0.86 ± 0.7 *	*p* < 0.005	0.73 ± 0.4	0.80 ± 0.4	—
**Romberg Index (EC/OE)**	1.46 ± 0.4	0.80 ± 0.6	—	1.41 ± 0.4	1.29 ± 0.4	—

## Data Availability

Data associated with the paper are not publicly available but are available from the corresponding author on reasonable request.

## Supplementary Materials

The following supporting information can be downloaded at: https://www.mdpi.com/article/10.3390/medicina62020300/s1, We have included the EVM machine and the stimulation points as additional material.

## References

[1] Peppe A., Paravati S., Baldassarre M.G., Bakdounes L., Spolaor F., Guiotto A., Pavan D., Sawacha Z., Bottino S., Clerici D. (2019). Proprioceptive Focal Stimulation (Equistasi^®^) May Improve the Quality of Gait in Middle-Moderate Parkinson’s Disease Patients. Double-Blind, Double-Dummy, Randomized, Crossover, Italian Multicentric Study. Front. Neurol..

[2] Ronconi G., Gatto D.M., Ariani M., Codazza S., Panunzio M., Coraci D., Ferrara P.E. (2024). Effects of focal muscle vibration on cervical pain in Parkinson’s disease patients: A pilot study. Eur. J. Transl. Myol..

[3] Liu W.Y., Tung T.H., Zhang C., Shi L. (2022). Systematic review for the prevention and management of falls and fear of falling in patients with Parkinson’s disease. Brain Behav..

[4] Paolucci T., Morone G., Fusco A., Giuliani M., Rosati E., Zangrando F., Saraceni V.M., Paolucci S., Iosa M. (2014). Effects of perceptive rehabilitation on balance control in patients with Parkinson’s disease. NeuroRehabilitation.

[5] Marazzi S., Kiper P., Palmer K., Agostini M., Turolla A. (2021). Effects of vibratory stimulation on balance and gait in Parkinson’s disease: A systematic review and meta-analysis. Eur. J. Phys. Rehabil. Med..

[6] Tan T., Almeida Q.J., Rahimi F. (2011). Proprioceptive deficits in Parkinson’s disease patients with freezing of gait. Neuroscience.

[7] Schrader C., Peschel T., Däuper J., Rollnik J., Dengler R., Kossev A. (2008). Changes in processing of proprioceptive information in Parkinson’s disease and multiple system atrophy. Clin. Neurophysiol..

[8] Leodori G., Santilli M., Modugno N., D’avino M., De Bartolo M.I., Fabbrini A., Rocchi L., Conte A., Fabbrini G., Belvisi D. (2023). Postural Instability and Risk of Falls in Patients with Parkinson’s Disease Treated with Deep Brain Stimulation: A Stabilometric Platform Study. Brain Sci..

[9] Radder D.L.M., de Lima A.L.S., Domingos J., Keus S.H.J., van Nimwegen M., Bloem B.R., de Vries N.M. (2020). Physiotherapy in Parkinson’s Disease: A Meta-Analysis of Present Treatment Modalities. Neurorehabilit. Neural Repair.

[10] Tinetti M.E. (1986). Performance-oriented assessment of mobility problems in elderly patients. J. Am. Geriatr. Soc..

[11] Tinetti M.E., Richman D., Powell L. (1990). Falls efficacy as a measure of fear of falling. J. Gerontol..

[12] Tinetti M.E. (2003). Clinical practice. Preventing falls in elderly persons. N. Engl. J. Med..

[13] Tjernström F., Björklund M., Malmström E.M. (2015). Romberg ratio in quiet stance posturography—Test to retest reliability. Gait Posture.

[14] Paolucci T., Iosa M., Morone G., Fratte M.D., Paolucci S., Saraceni V.M., Villani C. (2018). Romberg ratio coefficient in quiet stance and postural control in Parkinson’s disease. Neurol. Sci..

[15] Murillo N., Valls-Sole J., Vidal J., Opisso E., Medina J., Kumru H. (2014). Focal vibration in neurorehabilitation. Eur. J. Phys. Rehabil. Med..

[16] Seok J.W., Park S.R. (2025). Effects of Whole-Body, Local, and Modality-Specific Vibration Therapy on Gait in Parkinson’s Disease: A Systematic Review and Meta-Analysis. Biomedicines.

[17] van Heuvelen M.J.G., Rittweger J., Judex S., Sañudo B., Seixas A., Fuermaier A.B.M., Tucha O., Nyakas C., Marín P.J., Taiar R. (2021). Reporting guidelines for whole-body vibration studies in humans, animals and cell cultures: A consensus statement from an international group of experts. Biology.

[18] Guang H., Ji L., Shi Y. (2018). Focal Vibration Stretches Muscle Fibers by Producing Muscle Waves. IEEE Trans. Neural Syst. Rehabil. Eng..

[19] Filippi G.M., Rodio A., Fattorini L., Faralli M., Ricci G., Pettorossi V.E. (2023). Plastic changes induced by muscle focal vibration: A possible mechanism for long-term motor improvements. Front. Neurosci..

[20] Viganò A., Celletti C., Giuliani G., Jannini T.B., Marenco F., Maestrini I., Zumpano R., Vicenzini E., Altieri M., Camerota F. (2023). Focal Muscle Vibration (fMV) for Post-Stroke Motor Recovery: Multisite Neuroplasticity Induction, Timing of Intervention, Clinical Approaches, and Prospects from a Narrative Review. Vibration.

[21] Calderone A., Galasso S., De Nunzio A.M., Leo A., Balletta T., Quartarone A., Calabrò R.S. (2025). Exploring the Impact of Muscle Vibration Therapy in Neurologic Rehabilitation: A Systematic Review. Arch. Rehabil. Res. Clin. Transl..

[22] Giorgi F., Donati D., Platano D., Tedeschi R. (2024). Focal Vibration Therapy for Motor Deficits and Spasticity Management in Post-Stroke Rehabilitation. Brain Sci..

[23] Alashram A.R., Padua E., Romagnoli C., Raju M., Annino G. (2022). Clinical effectiveness of focal muscle vibration on gait and postural stability in individuals with neurological disorders: A systematic review. Physiother. Res. Int..

[24] Rafti D., Uzun A.B., Bodeanu L., Stanciu L.E., Popescu M.N., Iliescu M.G. (2025). The Potential of Focal Muscle Vibration Therapy in the Management of Parkinson’s Disease: A Systematic Review. J. Clin. Med..

[25] Camerota F., Celletti C., Suppa A., Galli M., Cimolin V., Filippi G.M., La Torre G., Albertini G., Stocchi F., De Pandis M.F. (2016). Focal Muscle Vibration Improves Gait in Parkinson’s Disease: A Pilot Randomized, Controlled Trial. Mov. Disord. Clin. Pract..

[26] Aprile I., Di Sipio E., Germanotta M., Simbolotti C., Padua L. (2016). Muscle focal vibration in healthy subjects: Evaluation of the effects on upper limb motor performance measured using a robotic device. Eur. J. Appl. Physiol..

[27] Filippi G.M., Di Stefano G., Paolucci T. (2023). Effects of focal muscle vibration on cervical pain in Parkinson’s disease: A clinical study. Clin. Rehabil..

[28] Clarke C.E., Patel S., Ives N., Rick C.E., Woolley R., Wheatley K., Walker M.F., Zhu S., Kandiyali R., Yao G. (2016). Clinical Effectiveness and Cost-Effectiveness of Physiotherapy and Occupational Therapy Versus No Therapy in Mild to Moderate Parkinson’s Disease: A Large Pragmatic Randomised Controlled Trial (PD REHAB).

[29] Rabey J.M., Korczyn A.D., Przuntek H., Kraus P.H., Klotz P., Korczyn A.D. (1995). The Hoehn and Yahr Rating Scale for Parkinson’s Disease. Instrumental Methods and Scoring in Extrapyramidal Disorders.

[30] Arevalo-Rodriguez I., Smailagic N., Roqué-Figuls M., Ciapponi A., Sanchez-Perez E., Giannakou A., Pedraza O.L., Bonfill Cosp X., Cullum S. (2021). Mini-Mental State Examination (MMSE) for the early detection of dementia in people with mild cognitive impairment (MCI). Cochrane Database Syst. Rev..

[31] Goetz C.G., Tilley B.C., Shaftman S.R., Stebbins G.T., Fahn S., Martinez-Martin P., Poewe W., Sampaio C., Stern M.B., Dodel R. (2008). Movement Disorder Society UPDRS Revision Task Force. Movement Disorder Society-sponsored revision of the Unified Parkinson’s Disease Rating Scale (MDS-UPDRS): Scale presentation and clinimetric testing results. Mov. Disord..

[32] Domingos J., Keus S.H., Dean J., de Vries N.M., Ferreira J.J., Bloem B.R. (2018). The european physiotherapy guideline for Parkinson’s disease: Implications for neurologists. J. Park. Dis..

[33] Ernst M., Folkerts A.K., Gollan R., Lieker E., Caro-Valenzuela J., Adams A., Cryns N., Monsef I., Dresen A., Roheger M. (2023). Physical exercise for people with Parkinson’s disease: A systematic review and network meta-analysis. Cochrane Database Syst. Rev..

[34] Tomlinson C.L., Herd C.P., Clarke C.E., Meek C., Patel S., Stowe R., Deane K.H.O., Shah L., Sackley C.M., Wheatley K. (2014). Physiotherapy for Parkinson’s disease: A comparison of techniques. Cochrane Database Syst. Rev..

[35] Hayes K.W., Johnson M.E. (2003). Measures of adult general performance tests: The Berg Balance Scale, Dynamic Gait Index (DGI), Gait Velocity, Physical Performance Test (PPT), Timed Chair Stand Test, Timed Up and Go, and Tinetti Performance-Oriented Mobility Assessment (POMA). Arthritis Rheum..

[36] Santamaría-Peláez M., González-Bernal J.J., Da Silva-González Á., Medina-Pascual E., Gentil-Gutiérrez A., Fernández-Solana J., Mielgo-Ayuso J., González-Santos J. (2023). Validity and Reliability of the Short Physical Performance Battery Tool in Institutionalized Spanish Older Adults. Nurs. Rep..

[37] McMichael K.A., Vander Bilt J., Lavery L., Rodriguez E., Ganguli M. (2008). Simple balance and mobility tests can assess falls risk when cognition is impaired. Geriatr. Nurs..

[38] Volpe D., Giantin M.G., Fasano A. (2014). A wearable proprioceptive stabilizer (Equistasi^®^) for rehabilitation of postural instability in Parkinson’s disease: A phase II randomized double-blind, double-dummy, controlled study. PLoS ONE.

[39] Spolaor F., Romanato M., Annamaria G., Peppe A., Bakdounes L., To D.-K., Volpe D., Sawacha Z. (2021). Relationship between muscular activity and postural control changes after proprioceptive focal stimulation (Equistasi^®^) in middle-moderate parkinson’s disease patients: An explorative study. Sensors.

[40] Brown M.C., Engberg I., Matthews P.B.C. (1967). The Relative Sensitivity to Vibration of Muscle Receptors of the Cat. J. Physiol..

[41] Catton W.T. (1970). Physiological Reviews Mechanoreceptor Function. http://www.physiology.org/journal/physrev.

[42] Rocchi L., Suppa A., Leodori G., Celletti C., Camerota F., Rothwell J., Berardelli A. (2018). Plasticity induced in the human spinal cord by focal muscle vibration. Front. Neurol..

[43] Viseux F.J.F. (2020). The sensory role of the sole of the foot: Review and update on clinical perspectives. Neurophysiol. Clin..

[44] Ferrara P.E., Gatto D.M., Codazza S., Zordan P., Stefinlongo G., Coraci D., Monaco M.R.L., Ricciardi D., Ronconi G. (2022). Effects of focal muscle vibration on gait and balance in Parkinson patients: Preliminary results. Appl. Sci..

[45] Allen N.E., Canning C.G., Almeida L.R.S., Bloem B.R., Keus S.H., Löfgren N., Nieuwboer A., Verheyden G.S., Yamato T.P., Sherrington C. (2022). Interventions for preventing falls in Parkinson’s disease. Cochrane Database Syst. Rev..

[46] Arias P., Chouza M., Vivas J., Cudeiro J. (2009). Effect of whole body vibration in Parkinson’s disease: A controlled study. Mov. Disord..

