# Supplementary material for: Effects of Focal Muscle Vibration on Static and Dynamic Balance in Patients with Parkinson’s Disease: Preliminary Results of a Retrospective Study
Source: Medicina (Kaunas). 2026 Feb 2;62(2):300. doi: 10.3390/medicina62020300 (PMC12942015; doi:10.3390/medicina62020300)

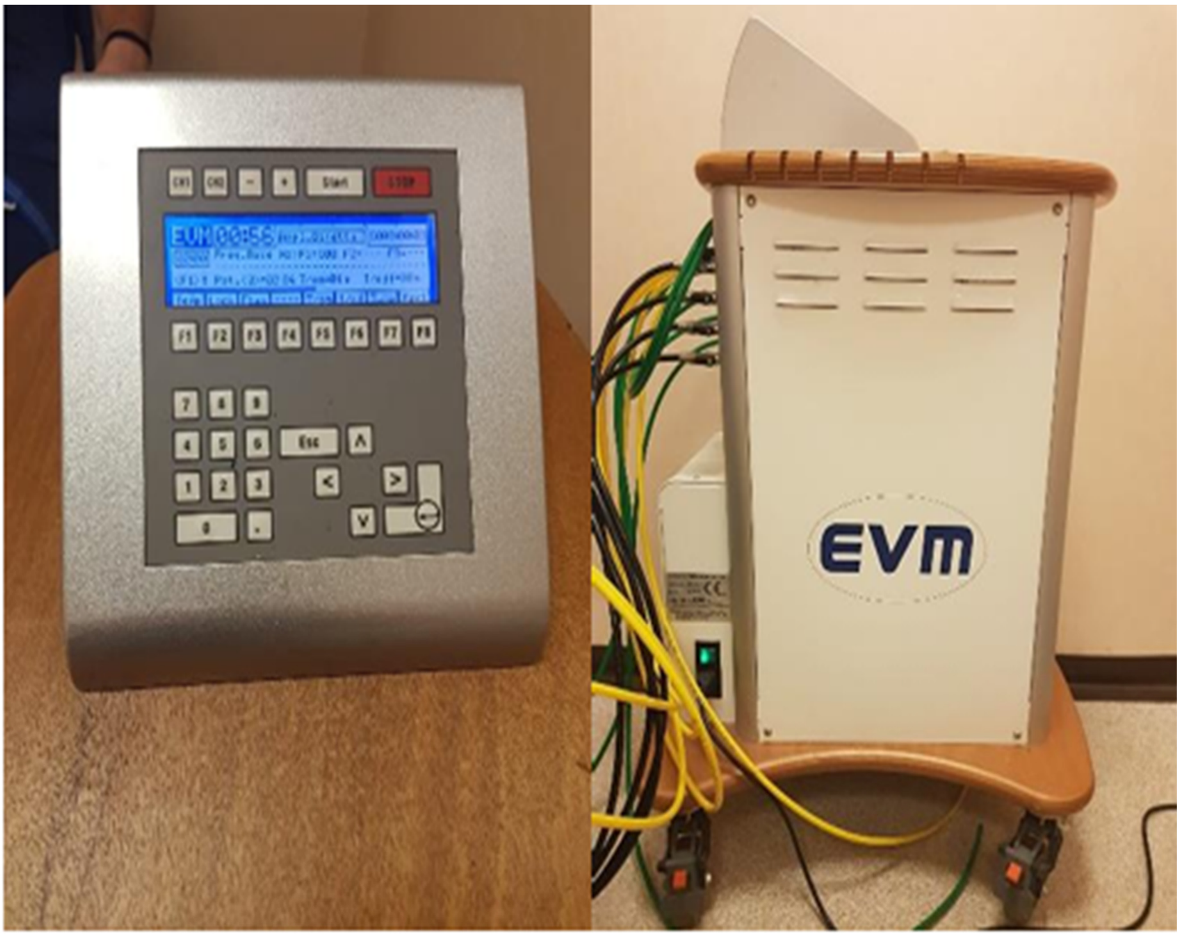

The EVM EVO (Muscle Vibratory Energy Evolution) device (Endomedica, Italy)

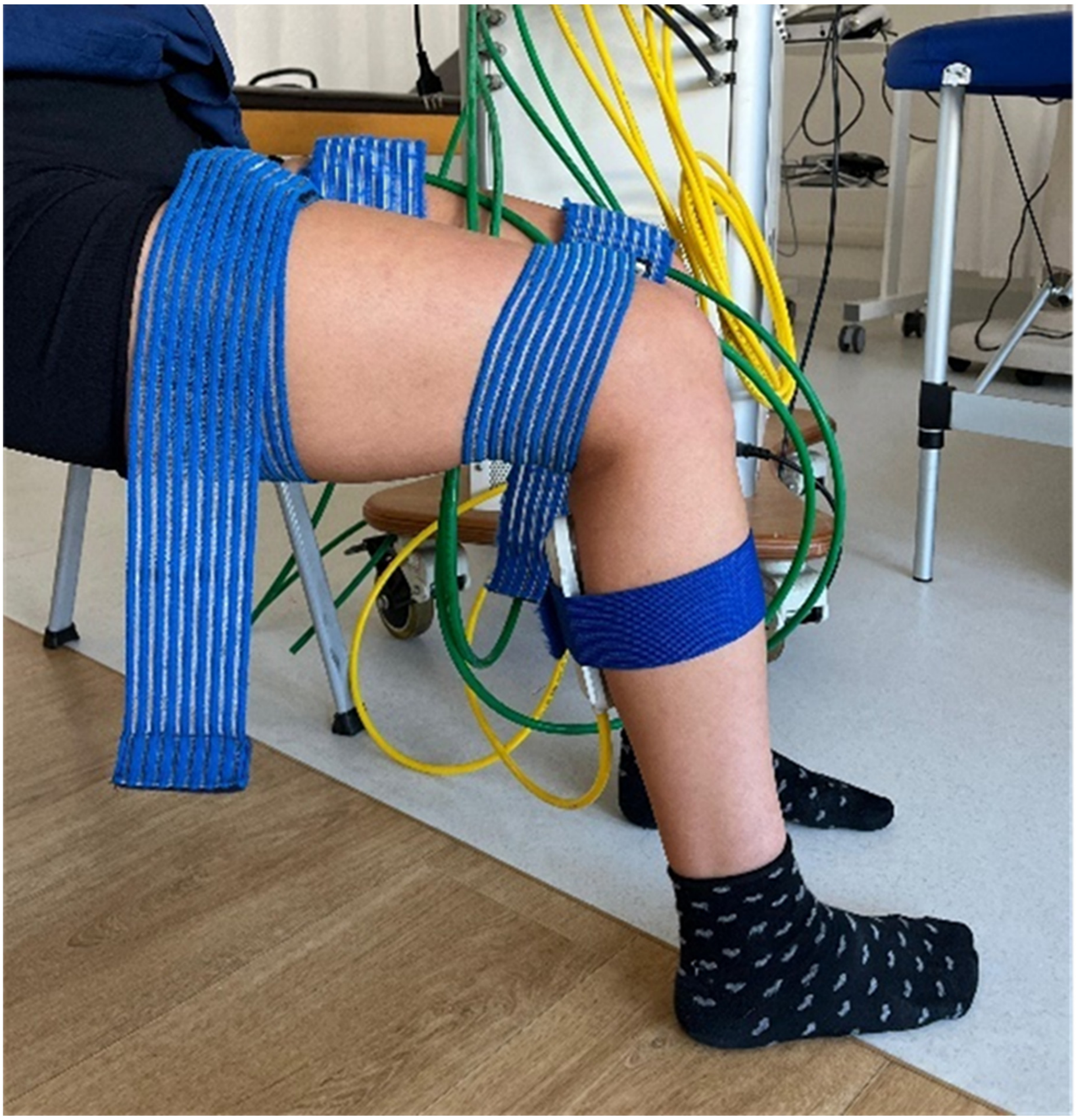

Treatment consisted of bilateral stimulation of the quadriceps femoris and gastrocnemius.

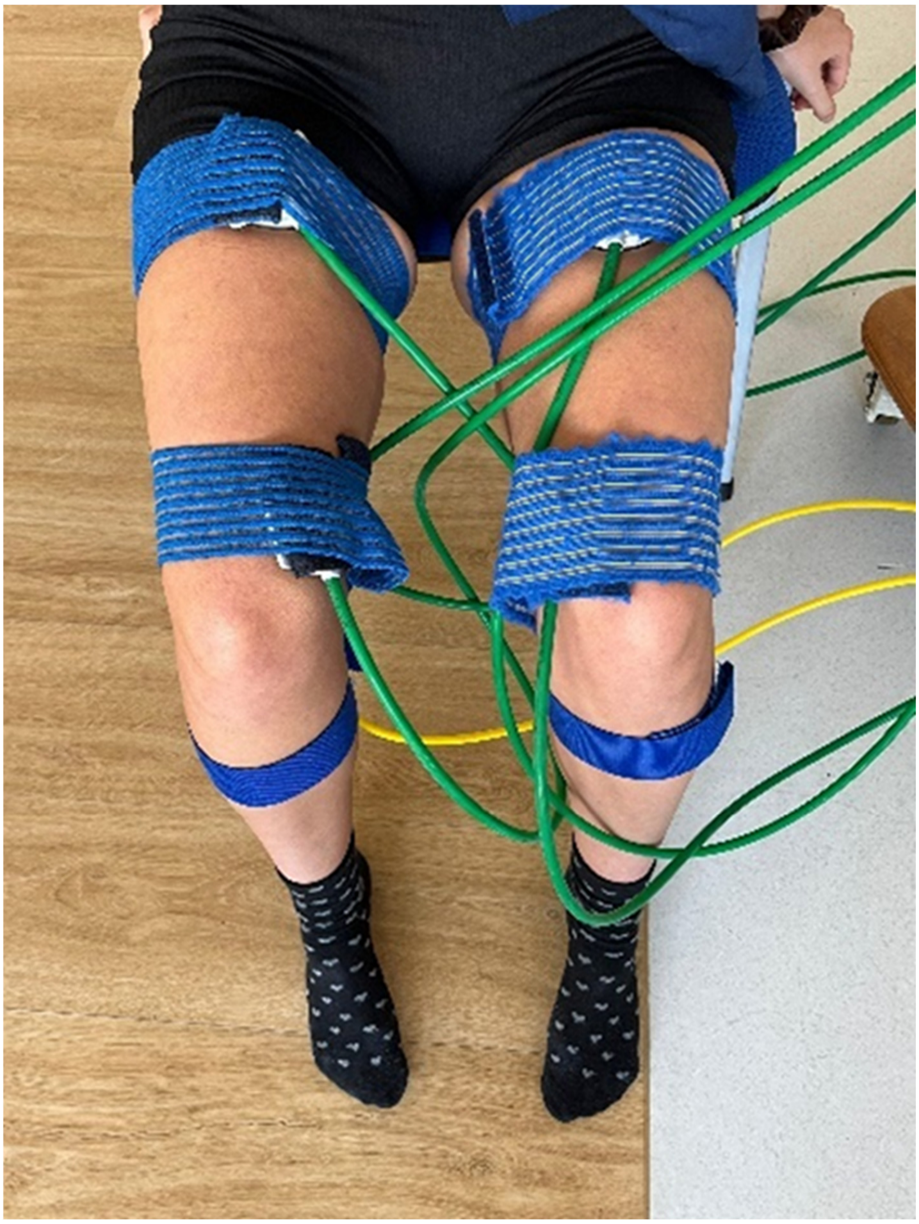

Supplement: Supplementary file 1 [file medicina-62-00300-s001.zip › medicina-4087388-supplementary.pdf]
